# Influencing factors for prevention of postpartum hemorrhage and early detection of childbearing women at risk in Northern Province of Rwanda: beneficiary and health worker perspectives

**DOI:** 10.1186/s12884-020-03389-7

**Published:** 2020-11-10

**Authors:** Oliva Bazirete, Manassé Nzayirambaho, Aline Umubyeyi, Marie Chantal Uwimana, Marilyn Evans

**Affiliations:** 1 College of Medicine and Health Sciences, University of Rwanda, 3286 Kigali, Rwanda; 2 University of Western Ontario, Arthur Labatt Family School of Nursing, 1151 Richmond St, London, ON N6A 3K7 Canada

**Keywords:** Postpartum hemorrhage, Early detection, Prevention, Influencing factors, Beneficiaries, Health workers

## Abstract

**Background:**

Reduction of maternal mortality and morbidity is a major global health priority. However, much remains unknown regarding factors associated with postpartum hemorrhage (PPH) among childbearing women in the Rwandan context. The aim of this study is to explore the influencing factors for prevention of PPH and early detection of childbearing women at risk as perceived by beneficiaries and health workers in the Northern Province of Rwanda.

**Methods:**

A qualitative descriptive exploratory study was drawn from a larger sequential exploratory-mixed methods study. Semi‐structured interviews were conducted with 11 women who experienced PPH within the 6 months prior to interview. In addition, focus group discussions were conducted with: women’s partners or close relatives (2 focus groups), community health workers (CHWs) in charge of maternal health (2 focus groups) and health care providers (3 focus groups). A socio ecological model was used to develop interview guides describing factors related to early detection and prevention of PPH in consideration of individual attributes, interpersonal, family and peer influences, intermediary determinants of health and structural determinants. The research protocol was approved by the University of Rwanda, College of Medicine and Health Sciences Institutional Ethics Review Board.

**Results:**

We generated four interrelated themes: (1) Meaning of PPH: beliefs, knowledge and understanding of PPH: (2) Organizational factors; (3) Caring and family involvement and (4) Perceived risk factors and barriers to PPH prevention. The findings from this study indicate that PPH was poorly understood by women and their partners. Family members and CHWs feel that their role for the prevention of PPH is to get the woman to the health facility on time. The main factors associated with PPH as described by participants were multiparty and retained placenta. Low socioeconomic status and delays to access health care were identified as the main barriers for the prevention of PPH.

**Conclusions:**

Addressing the identified factors could enhance early prevention of PPH among childbearing women. Placing emphasis on developing strategies for early detection of women at higher risk of developing PPH, continuous professional development of health care providers, developing educational materials for CHWs and family members could improve the prevention of PPH. Involvement of all levels of the health system was recommended for a proactive prevention of PPH. Further quantitative research, using case control design is warranted to develop a screening tool for early detection of PPH risk factors for a proactive prevention.

## Background

Maternal mortality remains a major challenge to the health system worldwide. The World Health Organization(WHO) [1] reports that every year, nearly 295 000 women die due to complications induced by the pregnancy and most of them are preventable or treatable. Maternal mortality from low and lower middle-income countries accounts for 94% [1]. Say et al. [2], highlight that more than 25% of these deaths result from postpartum bleeding, with almost 20% of all maternal deaths due to postpartum hemorrhage (PPH). In Rwanda, 70% of maternal deaths result from direct causes and postpartum bleeding is the leading direct cause of maternal death with 22.7% of all documented cases [3].

Primary PPH is commonly defined as a blood loss of 500 ml or more within 24 hours after birth [4, 5]. Though there have been concerted initiatives to address maternal mortality due to PPH, the issue remains a global burden [6]. The recent data from WHO [1], suggest that less than 50% of all births in several low income and lower-middle-income countries are assisted by a trained midwife, doctor or nurse while in most high-income and upper-middle-income countries, reports demonstrate that more than 90% of all births benefit from the presence of a skilled birth attendant. Factors related to provision of substandard care were identified for 61.1% of the maternal death cases in a study on maternal death audit in Rwanda [3].

As reported by evidence, effective implementation of guidelines based on evidence is deemed to prevent majority of PPH-related morbidity and mortality. WHO [7] provides recommendations for skilled health care providers and other stakeholders on the effective use of uterotonics for PPH prevention among women giving birth in hospital or community settings from high-income, middle-income or low-income countries. However, Raghavan et al. [8] affirm that routine uterotonic prophylaxis, such as, the use of Oxytocin as a gold standard uterotonic medication for PPH prevention, is not 100% effective in PPH prevention. Therefore, WHO [7] is urging different partners to review their respective national health policies and protocols on PPH prevention to reflect quality of care for women which involves timely and appropriate care to achieve desired outcomes consistent with professional knowledge and considering the preferences and aspirations of individual childbearing women and their families [9]. In addition, Prata et al. [10] highlight the need for every country to develop its own policies and programs specific to local context and incorporating a variety of approaches to prevent and address PPH challenges.

Different ways to prevent PPH fall under the widely used Essential Public Health Operations (EPHO) that different countries with the leadership of WHO can adapt and work on together, to assess and plan for sustainable public health services and capacities [11]. The EPHO-five, focuses on prevention of disease through actions taken across the three levels of disease prevention: primary, secondary and tertiary levels. Almost all the actions from these levels of prevention, are within the roles and responsibilities of health care providers in primary care, hospitals and community services environments [11]. All the actions done to prevent risks before to onset of a disease (PPH in the case of the present paper), are proactive and constitute primary prevention. A recent qualitative systematic review conducted by Finlayson et al. [6] appraised evidence about the views and experiences of women and healthcare providers on interventions taken to prevent PPH at different prevention levels. The review generated considerable emphasis on contextual factors that contribute to successful implementation of strategies for PPH prevention, especially factors associated with sufficient resources and effective implementation by competent, suitably trained providers [6].

There is cause to consider that the investigation of factors influencing early detection of women at risk at all levels of PPH prevention from different perspectives is crucial to enhance quality of care during childbearing period. Striking factors at different levels of service delivery have been found to be critical in shaping participants’ experience for the implementation of obstetric hemorrhage initiative in Florida [12]. Furthermore, the study of Semasaka et al. [13] on self-reported pregnancy-related health problems and self-rated health status in postpartum Rwandan women observed poor sexual and reproductive health care and recommended that particular attention be given to determinants of poor quality care and to early prevention of related complications. Poverty, distance to facilities, lack of information, inadequate and poor quality services, and cultural beliefs and practices are reported by WHO [1] as different categories of main factors that prevent women from getting access to care during pregnancy and childbirth.

Lack of information among clients and their partners before, during and after the PPH event was the main obstacle reported by Woiski et al. [14] for high quality PPH-care identified by patients, while health care providers expressed lack of clarity of the guidelines, lack of knowledge and poor communication within teams as hindering factors. Therefore to improve quality of care provided to women for prevention of PPH, an in-depth analysis from different perspectives ,identifying influencing factors for delivery of quality PPH-care will provide necessary information for implementing a strategy to improve care [15]. Currently, little is known in Rwanda about key factors that could influence early detection of women at risk of PPH from the perspectives of patients and family members, the community and health care professionals. This study aims to explore influencing factors for prevention of PPH and early detection of at risk childbearing women as perceived by beneficiaries and health workers in the Northern Province of Rwanda.

## Methods

### Design

As part of a larger exploratory sequential mixed-methods study, we undertook phase one of the study with a qualitative descriptive design to develop a rich description of the phenomenon under study [16, 17]. This design was used to uncover daily participants’ experiences by remaining close to described or observed events [18]. To explore influencing factors for delivering PPH proactive preventive care from different perspectives, a social determinants approach to maternal deaths [19] and Social Ecological Model (SEM) [20] were used.

### Setting

**Fig. 1:**
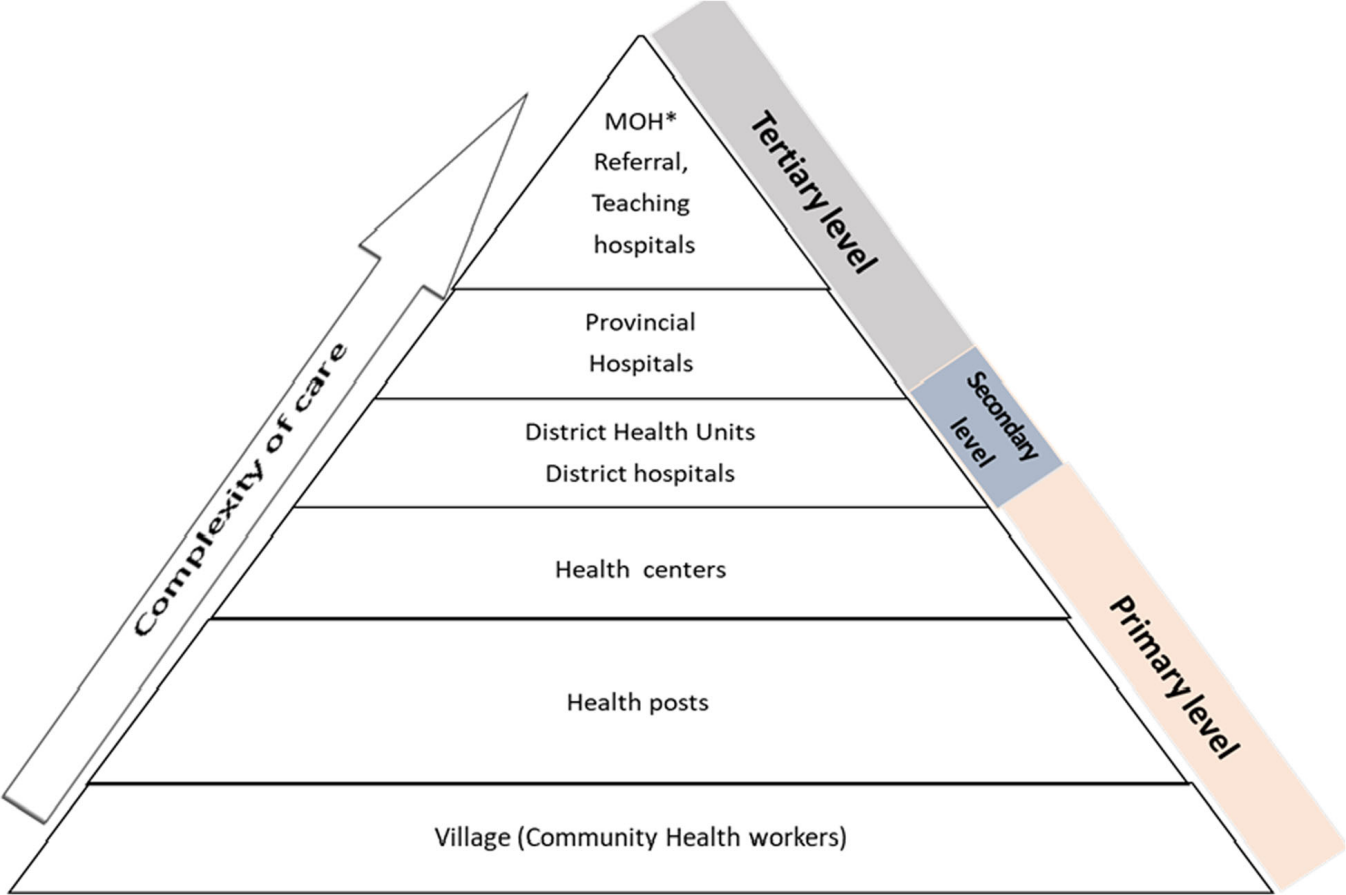
Health care system in Rwanda. MOH*: Ministry of Health

The healthcare system in Rwanda reaches from the community to the national referral hospitals [21]. As illustrated by the Fig. 1, there are three levels of the healthcare system in Rwanda: (1) community health centers and health posts which constitute the primary level of healthcare, (2) district hospitals operating at the secondary level of healthcare, and (3) provincial referral hospitals, national referral hospitals and University Teaching Hospitals, serving as the tertiary and highest level of healthcare [21, 22].

Recently, Rwandan Ministry of Health established a new level of health facility which is in between district hospital and health center, to relieve the challenge of delays in referral of obstetric cases and overcrowding at district hospitals. These facilities are called medicalized health centers [23].

The present study was conducted at primary and secondary levels of the health system in Rwanda. We reached out to the community by involving community members and community health workers (CHWs) in charge of maternal health. This study was conducted in three health facilities of the Northern Province of Rwanda. We included one health center, one medicalized health center and one district hospital. The selection criteria we used to choose facilities to be visited included their level of performance in maternal and newborn health, location (urban versus rural), and geographical accessibility of the health facilities to clients. The study sites were selected by the principal investigator and validated by the research committee. The selected district hospital serves a population of 444 387 in its catchment area, which also includes 24 health centers [24]. The Northern Province of Rwanda was purposively chosen for being in a rural area where some health centers are hard to access, and for its low uptake of antenatal and postnatal services among childbearing women [24].

### Participants and recruitment

Fifteen women were purposively selected for having experienced PPH within the six months immediately prior to data collection, being willing to participate, and being over 18 years old. The research team, in collaboration with the head of maternity at the facility, identified PPH cases from the birth register. For women who were discharged from the health facilities, the CHWs in charge of maternal health assisted to connect the research team with them for recruitment from the villages. Women who were still in hospital were given a verbal invitation by the researcher to participate. Women and their partners or close relatives who agreed to participate were given an appointment by the researcher for an interview at the nearest health facility. The inclusion criteria for relatives included being her husband or a close relative who was with her when PPH occurred. The final sample size of eleven women was reached when it was determined no new themes were emerging from the interviews and it was judged that sufficient data had been collected to address the study’s purpose [16]. Ten close relatives to the women from two health facilities responded to our invitation and were willing to participate (eight husbands and two close female relatives).

CHWs in charge of maternal health living in the same village as the woman who experienced PPH were also invited to participate in this study since they are in control for not only maternal health but also for neonatal health in their respective villages. CHWs register women of reproductive age, encourage them to use maternal care services, go along with clients in labor from villages to the nearest health facility to be assisted by skilled birth attendants during labor and delivery [25]. The CHWs who participated in the present study were identified through the CHW coordinator who is a full time employee at the health facility. The researcher made a phone call to fourteen CHWs who were eligible, inviting them to participate in the study. Eleven female CHWs from the two health facilities responded to the invitation and were sent a text message specifying the venue and time for the focus group interview.

A total of twenty-five health care providers working in maternity units of selected health facilities were also invited to participate, of whom fourteen (10 nurses, 3 midwives, 1 medical doctor) were eligible to participate in a focus group discussion (FGD). Correspondence to potential participants was in Kinyarwanda language. Participants were recruited using email or telephone messages. Inclusion criteria included: being a full-time and health care provider with at least one year’s experience of working in maternity, and ability to speak and read in either English or Kinyarwanda.

In this study women and their relatives are considered as beneficiaries while health care professionals and CHWs in charge of maternal health are all considered as health workers. The research team consisted of members with expertise in qualitative research methodology, maternal health and health care. Apart from one research team member, all were female.

### Data collection

The research team developed semi-structured interview guides in both English and Kinyarwanda, including one for each category of in-depth interviews (IDIs) and Focus Group Discussions (FGDs). The interview guides were translated back and forth by an independent professional translator, to confirm that the meaning and content of the questions of the original copy had not changed during the translation process. Verification of the translated instrument was also done by the research team to ensure its validity. The interview guide questions focused on five interrelated levels of SEM to facilitate identification and description of potential PPH influencing factors: individual, interpersonal, community, organizational, and policy/enabling environment [20]. Demographic data of participants was obtained using a demographic form during individual interviews. All participants chose to be interviewed in Kinyarwanda. The principal investigator conducted IDIs and FGDs. As the researcher is a Rwandan national, there were no language or cultural barriers with participants.

Pretesting of the interview guides was conducted to improve validity in data collection procedures and interpretation of findings [26]. The pretest was done with participants not included in this study but sharing same inclusion criteria. This process assisted to determine the relevancy and appropriateness of the questions being asked, to assess wording and identify any difficulties. The pretest results indicated that conducting IDIs while a woman was still hospitalized, was not conducive for a free and open discussion. In fact, women were reluctant to talk about issues pertaining to their relationships with health care providers. Therefore, for the women who were still hospitalized at the health facilities included in this study, IDIs were scheduled on the day of their discharge.

Prior to voluntarily participating in the study, participants were informed about the purpose of the study and provided with a letter of information and consent form for their signature. Anonymity and confidentiality were observed throughout the conduct of the study. After obtaining participants’ informed consent, all interviews were digitally audio-recorded with participants’ permission and transcribed verbatim by the researcher. Data collection took place over a period of three months from December 2018 to February 2019 in meeting rooms of the three selected health facilities.

First, the researcher conducted one-to-one in-depth interviews with eleven women who had experienced PPH. Then, FGDs took place with the three groups of participants: (1) two FGDs with the partners and close relatives of the women, (2) two FGDs with CHWs and (3) three FGDs with health care providers. The duration of IDIs ranged between 45 and 60 minutes, while the FGDs lasted 45 to 90 minutes. To produce an in-depth understanding of early detection of women at risk of PPH and its prevention in relation to different contexts, field notes were taken by the researcher during and after data collection to capture all respondents’ nonverbal communications and other important information from the researcher observations.

The combination of IDIs and FGDs was used to seek data completeness [27] in this study. Each method (IDIs and FGDs) revealed different information about prevention of PPH and contributed to a more comprehensive understanding. Integration of data involved moving back and forth between the data sets to discover data convergence and complementarity.

After the number of IDIs and FGDs described above were completed, the participants’ responses had become repetitive, therefore it was determined that data saturation had been reached and recruitment ceased. The Table 1 illustrates number of participants in individual interviews and in focus groups discussions.

**Table 1:** Methods of data collection and participants in the qualitative study

Data collection methods	Participants	Number of IDIs/ FGDs	Number of Participants
**IDIs**	Women	11	11
**FGDs**	Health care providers	3	14
	Husbands/close relatives	2	10
	Community health workers	2	11

### Data analysis

Data analysis was concurrently undertaken with data collection and was initiated after the completion of the first interview. NVivo Pro Version 12 was used to help organize the data for further analysis. To analyze findings from the present study, we used the six steps of inductive thematic analysis [28, 29]. As described by Braun and Clarke [28] we focused on interpreting and explaining what the study participants shared. Throughout this process the researcher considers whether the identified themes work in the context of the entire data set and refines the developed themes to ensure they are coherent and distinct from each other [30]. The transcripts were read while listening to the audio recordings to ensure accuracy and completeness.

First, we read and re read the transcripts to become familiar with what was stated and to be immersed in the data, noting initial analytic observations. Second, we engaged in open line by line coding and assigned preliminary codes to the data in order to describe the content with interesting features across the entire data set. A coding guide was developed, consisting of all the codes or labels from the transcripts. Third, we proceeded to group familiar codes into preliminary themes which depicted the same ideas or concepts. The themes were discussed and agreed by the research team members through consensus. Ongoing analysis helped to refine the specifics of the themes then clear definitions and names for each theme were created. Finally we produced a report [29] on influencing factors for PPH prevention care from different perspectives. The research team worked in close collaboration throughout the data analysis process to discuss the codes and preliminary themes, and come to a consensus of the final emergent themes. Verbatim quotes were selected from the transcripts to illustrate main themes.

### Data quality

To ensure rigor [31] of the present qualitative study, trustworthiness was established by observing the criteria suggested by Lincoln and Guba [32]: credibility, transferability, dependability and confirmability.

To prepare for data collection, the interview guides were developed by the principal investigator after a literature search and critical discussion with the research committee members. The interview guide was initially pilot tested with three participants who are not included in the present study. For the credibility of data, we used investigator triangulation. For data quality checkup and consistency, reliability [33] was observed. Two transcripts from IDIs and one transcript from a FGDs were randomly selected by the principal investigator and shared with research team members to ensure that findings are based on participants’ responses rather than on researcher’s own preconceptions. The resulting comments were discussed and final decisions on codes and themes were made by group consensus. We also involved an independent researcher, with a master’s degree in public health to analyze a set of data while the principal investigator who conducted the interviews verified the consistency and fit of analyzed data with the original transcripts and audio records. Credibility of data was also ensured by data triangulation by using different methods and varying sources to collect data to develop a comprehensive understanding of factors affecting PPH prevention. IDIs and FGDs were conducted with different groups of people believed to have information about the topic under study. Data were gathered from three health facilities from the Northern Province of Rwanda offering different levels of health services to ensure greater representation of participants from various contexts and experiences. One medical doctor in charge of maternity unit was recruited to ensure that a wide range of insights were gained about the phenomenon.

During data collection and analysis, to account for personal bias and maintain objectivity, the researcher used journal writing to highlight the researcher’s reflections on the research in progress. A verbal check was also made by the researcher during and at the end of each interview, asking the participant to confirm whether the researcher’s understanding of the provided information aligned with what the participant had meant to say. After data analysis, three participants (one from IDIs and two from FGDs) were contacted with a phone call to obtain feedback on the generated themes and categories.

To ensure dependability, an audit trail was maintained to record the details of data collection, data analysis and the decisions made throughout the research process that led to the findings.

This study was presented and assessed by the Institution Review Board at the College of Medicine and Health Sciences, University of Rwanda, and approval (No 313/CMHS IRB/2018) to carry out the study was granted in accordance with the applicable rules concerning the review of research ethics committee and informed consent.

## Results

A total number of 46 informants participated in individual in-depth interviews (11 women) and FGDs (7 groups involving a total of 35 participants). To ensure confidentiality of study participants, they are identified as follows: W1, 2, 3, ….. (women); HCP1, 2, 3, ….. (health care professionals), CHW1, 2, 3, ….. (Community Heath Workers), R1, 2, 3... (relatives).

### Characteristics of participants in the individual in-depth interviews

Of the 11 women interviewed, six were aged between 20 and 34 years, and five were aged between 35 and 43 years. The majority of the women (*n* = 7) were in the range between parity 1–4 while four were parity 5 or above. Eight women lived in an area where they experienced difficulties to access the nearest health facility and six recalled having received Oxytocin after delivery (the other five did not know).

### Characteristics of participants in the FGDs

The minimum number of participants in an FGD was four and the maximum six. Of the 14 participants in the health care provider FGDs, nine were male and five were female; 10 were nurses, 3 were midwives and 1 was a medical doctor. They had between 2 and 35 years of experience working in reproductive health care. Of the 10 participants in the relatives FGDs, eight were husbands and two were relatives. Eleven CHW’s in charge of maternal health participated in the two FGDs. All CHWs who participated in this study were female, reflecting the reality in Rwanda that CHWs responsible for maternal health care in community settings are all female.

### Factors influencing the quality of PPH prevention

Four interrelated themes that described the factors influencing PPH prevention care were identified: (1) The meaning of PPH: personal beliefs, knowledge and understanding (2) Organizational factors (3) Caring and family involvement and (4) Perceived risk factors and barriers to PPH prevention. These themes included several sub-themes, which will be described in the following sections.

#### The meaning of PPH: personal beliefs, knowledge and understanding

This theme incorporates the way in which women, their partners or close relatives, CHWs in charge of maternal health, and health care providers think about PPH, and what it means in this context. It highlights the variety of beliefs, and clinical understanding related to the nature and prevention of PPH. It also describes need for more information about PPH, in terms of limited knowledge from a beneficiary’s perspective and additional training needs from a healthcare provider perspective.

The health care providers working in health facilities defined PPH as blood loss more than 500 ml within the first hour after birth and the quantity of blood loss was described as being visually estimated. Most CHWs described recognizing PPH in a woman when she “*changes the sanitary pad two times or more within the first hour”* after childbirth. But the majority of the women participants described bleeding after childbirth as “*not well known”* but an unusual blood loss after birth is a condition that needs to be resolved in a hospital setting. When asked about PPH one woman commented,

*“...I really don’t know what is it but what I know from myself, I delivered my child after a while I felt like I was sleeping in a basin of water, was full of blood all over, was feeling dizzy and told the nurse that I was uncomfortable the whole body, after that I did not know what was the next, and I woke up after being transferred in another referral hospital” (****W1).***

PPH was also described by relatives as coming *“unexpectedly”.* One participant explained that every woman is *“candidate”* to PPH and need to be prepared: *“When I try to look through it I assume that this problem happens unexpectedly and my conclusion is that every mother is a candidate, that is why all women must be prepared whether they are rich or poor”****(R12).***

Many health care providers mentioned that PPH has been associated with common causes like uterine tonicity, retained tissues after birth, tearing and trauma of genital organs during birth, and coagulopathy problems. Many participants felt the condition was associated with some cultural beliefs ,such as, hard manual labor performed by the woman, poison in the village, and traditional medicine, which may delay women seeking appropriate care: *“Now in the village they like to say that the problem is associated to poisons and a woman may go to the traditional doctor which can be the reason to be late to reach the health facility and may lead to those problems of bleeding”****(HCP22).***

Participants revealed that there is a cultural practice of *“hiding”* a complication that might happen during childbearing. Close relatives indicated that such hiding might be associated with lack of awareness about PPH in the community as the condition is believed to be associated to poison: *“what I saw in many Rwandans is that they try to hide that they have had a complication afterbirth. I think this might be associated to lack of awareness of the causes as some might link it to poison”****(R17***). Participants mentioned that family members have a great influence in forcing women to follow what they believe in, such as the use of traditional medicines. One participant mentioned cultural beliefs about care during childbirth might be contradictory to the woman’s own knowledge on PPH. “..*for example a woman can be aware of the signs that can lead to PPH but her mother-in-law oblige her to take traditional medicines telling her that if her grandchild faces a problem because she did not take those traditional medicines she will be accountable and will explain it”****(HCP29).***

Participants shared their desire for information to improve their knowledge on PPH prevention. The women and their relatives revealed that CHWs in charge of maternal health in their village educate women and their families about abnormal signs (in this case, participants talked about severe headaches, fever and bleeding) during pregnancy and in postpartum period. Participants stated that CHWs encourage pregnant women in their villages to go to the health facility for antenatal consultation and delivery. They suggested to have local leaders, for example administrative leaders of the local village, educated about PPH as they are close to the population in the village. “…...*I can suggest that all leaders at the villages’ level can take this as their duty and I think this can contribute a lot to prevention of some maternal health problems*.” **(R21).**

The health care providers and CHWs expressed the need for continuous training on PPH as many of them change the workplace. They mentioned that new staff might not be aware of updated protocols in PPH prevention. The health care providers working in antenatal clinics stated that when they are well informed about PPH and its risk factors, they are able to effectively teach and help women gain knowledge about the signs and symptoms of PPH and what actions to take. CHWs in particular said that they wanted to be adequately trained on some procedures like assisting home deliveries so they are able to provide care in case a woman delivers in the community before reaching the health facility. One CHW explained: “…*as a community health worker who meet women with PPH before reaching the health center, and as you know it is most of the times difficult for them to get transport, my suggestion is that they can train us on basic practices, like home deliveries, and delivery of the placenta so that the woman reaches the health center after being basically treated*”.

#### Organizational factors influencing PPH prevention

This theme highlights some of the organizational factors that influence PPH prevention. Healthcare providers identified factors associated with some existing PPH prevention policies in Rwanda. The majority of participants felt that adequate resources were a necessary factor in prevention, as well as collaboration across the health system structure.

Health care providers stated that teaching women about PPH and prevention strategies is a required and expected part of maternal health care.

*“……. Our Ministry of Health always encourage the health providers at the hospitals and health centers to teach pregnant women to go for the pregnancy checkup and to give birth in a hospital setting and I think this contributes to the prevention of PPH…..”****( HCP13).***

CHWs expressed that their role is to educate woman about risk factors and “*get her to the health facility”* when she is approaching the expected date of delivery to receive care from a skilled birth attendant. CHWs mentioned their main functions in the event a woman gives birth at home or in the community to prevent PPH. If the woman gives birth at home or on the way to the health facility, the policy of task shifting allows CHWs to provide Misoprostol to the woman after delivery to prevent PPH. *“..when a woman gives birth at home or before reaching the hospital we give her the misoprostol which reduces the hemorrhage, then we take her to the health providers who orders her to take enough rest. For us we use to provide advices to the women and the trainings that we received help them (CHW36).*

In addition to policies regarding maternal care, participants expressed that limited human and material resources and lack of continuity of care across the health system impact PPH prevention. The shortage of qualified health professional in maternity care was highlighted as a challenge by all participants. Participants stated that having “*specialized health professionals”* in health care settings would contribute to the reduction of PPH cases. Health care providers revealed that having only a small number of knowledgeable staff on a shift creates problems “*to follow up properly*” women every fifteen minutes after birth, and they do not have time to effectively teach mothers about factors that may lead to PPH. Relatives of the women mentioned that health care providers’ heavy work load may hamper recognizing a client who is bleeding after birth:

“… *all levels may influence our women to bleed. The health care provider may be overwhelmed because of many patients when she is one or two on a shift, it is hard for her/him to manage. For example my wife gave birth without any complication but by accident she was damaged which caused her to bleed, I could not say that it is the misunderstandings instead it was the problem of health personnel*” (**R16**).

Though health care providers were aware of the recommendation to administer injectable oxytocin for the management of third stage of labor to prevent PPH, many stated they were not confident about its effectiveness because of the heat sensitivity of the medicine. The lack of refrigerators in maternity units to store oxytocin was also highlighted as their main challenge for quality prevention of PPH.

***“…****the injectable oxytocin we use is the one to be kept in the fridge but all maternities in the health centers or the hospital do not have a fridge to keep the oxytocin, it is kept in the general pharmacy which will prevent us to give the oxytocin on time and with appropriate temperature...”****(HCP35).***

Furthermore, the majority of participants mentioned the importance of information sharing for the continuity of care and a proper follow up of women across the health system from the community to the district hospital. When there is a woman in labor or with another obstetrical problem in the community, the CHWs, through a system of “*rapid SMS”*, use their cell phones to call health providers at the health facility to send the ambulance. Women and their relatives affirmed that this is a good collaboration between the health facility and the CHWs, although sometimes there is delay in sending the ambulance. However, women identified the need for getting accurate information about PPH during pregnancy, delivery and the postpartum period so that they can make informed decisions regarding when to seek follow up care. The health care providers mentioned that the client’s health information related to her pregnancy is not well shared from the antenatal clinic of the health center to the maternity setting where the woman gives birth, which can further impede the recognition of clients at risk of PPH.

*“Most of the signs and symptoms discovered during antenatal consultation remain in the clinic, a woman does not have that information, what is only written on her file is to come early and give birth at the health center”****(HCP29).***

Participants mentioned that for a proper prevention of PPH the awareness should be enhanced in the health system so as to ease the identification of risk factors as early as possible by means of regular checkup of well-informed women before and after delivery.

#### Caring and family involvement

This theme reveals personal qualities, role expectations and clinical skills valued by women and their relatives, CHWs in charge of maternal health and health care providers during their interactions to prevent PPH. It also highlights some disrespectful practices that women experienced while seeking health care.

Participants discussed family involvement in their decision-making to prevent PPH. The women mentioned feeling dependent upon family members for assistance during pregnancy and childbirth. *“The family help me in not doing heavy activities and not being stressed… I first inform the person I live with, here I mean my husband, then we take a decision to go to the hospital because they are the right people to help me prevent against PPH.”***(W2)**.

The partners to women expressed the feeling of “*being less helpful*” to women in terms of PPH prevention. They explained their lack of knowledge about PPH which affects their ability to make informed decision on the health conditions of their wives. They described their main role as to “*get the woman to the health facility*” to be assessed and treated by qualified health professionals. However, CHWs argued that prevention of PPH should start in the nuclear family, with parents teaching their young daughters about how to prevent. *“A family has to be the first one to teach their young daughters to prevent early pregnancies which may lead to PPH, and to have that discussion in their home.”****(CHW43)***.

The women and their relatives view the role of CHWs in charge of maternal health in their community as their *“parent”*, who will closely follow up pregnant women and report to the health facility as needed. They also pointed out that CHWs have insufficient knowledge about PPH and preventive strategies to provide enough information to community members. *“Community health workers are available but we do not discuss about that issue of bleeding. They accomplish well their tasks but I think they do not know much about PPH so that they can teach us too about it”****(W10).***

Regarding the care provided by health care providers, women’s relatives recognize the busy work of health professionals. Beside the busy work, relatives of women described an issue of lack of focus by health care providers while providing services. They reported that some of them used to be busy on their cell phones making personal calls which prevented them from providing quick and timely healthcare services to women. Relatedly, they expressed their appreciation about the government’s policy which was put in place in response to this problem. For these relatives, the government’s response is viewed as one of the ways to improve recognition of women at risk of PPH.

*“There are things that the government has changed according to the way we were used to be given medical services like that thing of stopping cell phones at hospitals changed a lot things and we are so thankful. Before when we needed assistance from them, we used to find them busy on phone.”****(R20).***

Women and their relatives expressed feelings of frustration and anxiety when they encounter angry and irritable health care providers while receiving care. They described poor patient-health provider relationships can pose a communication barrier for the prevention of PPH. As one relative stated:

*…there are some problems we face at hospitals where we find doctors or other medical professions who are always angry or with bad services and you will realize that some patients are not comfortable and are fearing to tell everything to the health care provider. There are people who can look at you and you have fear to express yourself….****(R19).***

#### Perceived risk factors to PPH prevention

This theme describes various risk factors associated with PPH which participants describe as antepartum and intrapartum risk factors. They also stated that the socioeconomic status of the family and delays to receiving health care are factors affecting access to quality care for PPH prevention. Participants highlighted that knowledge and consideration of these risk factors can contribute greatly to timely prevention of PPH.

Health care providers mentioned that the “*knowing of pregnant women with predisposing risk to PPH*” would contribute to PPH prevention. As presented in the previous themes, participants expressed that the lack of knowledge and insufficient information sharing across all levels of care is a barrier to the recognition of the clients at risk of PPH and hinders effective and timely PPH prevention. The risk factors were described as non-use of family planning methods leading to frequent birthing, history of PPH, retained placenta, and tearing/trauma of genital organs. In case of trauma or lacerations of genital organs during birth, women and their relatives mentioned that birth attendants “*damage the woman’s internal tissues”*. Some risk factors were thought by women and relatives to be associated with religious beliefs where some people consider “*use of family planning as sinful”* and as a result give birth frequently without birth spacing. Women and their relatives commented that giving birth at home heightens the risk of complications such as retained placenta or tears of genital organs resulting in PPH.

*“...my last born was born at home but the placenta remained inside and I bled and bled a lot so they took me to the health center. I recovered my consciousness when I realized that I was lying on the bed of the health center…”****(W 11).***

Relatives of women as well as health care providers also expressed the view that poverty and poor nutrition exposes the woman to developing PPH. “*What I can add is that the challenge the society meet is the poverty because if a pregnant woman does not eat a balanced diet when she is pregnant, she may have post-partum hemorrhage after giving birth*”. ***(R13****)*

Participants also highlighted barriers related to delays to seeking care which prevent women from receiving quality services for the prevention of PPH. The socioeconomic status of the family was mentioned by the majority of all participants to adversely impact PPH prevention, particularly, poverty. CHWs highlighted that poor families experience the challenge of not being able to afford to buy basic food or to seek care at the health facility, which is believed to increase their risk for PPH.

*“There is a problem of poverty like people in the first category are our big challenge because they are the ones who live with malnutrition problems and give birth frequently, they tell you their problems at a later stage when the woman cannot even sit on a motorcycle and we pay for their transport (****CHW38).***

Family conflict was also expressed as a challenge associated with socioeconomic conditions. Health care providers revealed that families living with conflict may be less likely to make good decisions regarding health and pay for medical insurance, hence they don’t access medical services on time contributing to childbearing complications.

“*Families can miss the insurance and you may find a husband in a family who is a drunker or cheating on his wife. When it comes to go to hospital the wife miss someone to accompany her and then she chooses to stay home instead of paying a motorcycle for transportation, a community health worker can recognize this situation late when a woman is in a bad situation, that is how poverty is still a barrier in our zone” (****HCP29****).*

Participants stated that the shortage of qualified staff leads to the delay of proper follow up of childbearing women especially those at risk to develop PPH. This has been expressed as a “*delay to attend a case*” which might mean that signs of an emergency are missed as expressed by a CHW: *“A health provider might be working alone in maternity and she has assisted a woman to give birth thereafter, she might be called by other women in labor to look after them, and meanwhile the lady who just gave birth is left alone, no one is there to provide follow up. In this case the woman may be at risk of bleeding, then bleed and the health care provider will delay to attend the case, no one will know…”***(CHW39**).

Geography and location of health facilities were mentioned by participants as a challenges to PPH prevention. Many of the women commented on the delay associated to “*the location of some health facilities is also mattering because we live in high hilled lands* “and people resort to taking motorcycles in case the ambulance takes a long time to reach them. The delay to transfer the woman from a health center to a district hospital has been also expressed as a risk to a woman to have her health status complicated:

*“… there is a problem of a delaying decision making when a woman is at the health center or hospital, they may delay to take decision to refer her at night while she has been there for a day bleeding and when she reaches the hospital they may try to intervene while it is no longer possible…” (****HCP34)***.

To address the factors influencing PPH, participants recommended placing an emphasis on prevention strategies pre-conception and antenatally.

## Discussion

This study explored influencing factors for prevention of PPH and early detection of childbearing women at risk as perceived by beneficiaries (childbearing women and their close relatives) and health workers (CHWs in charge of maternal health and health care providers) in the Northern Province of Rwanda.

### Beneficiary perspectives

The results from the present study suggest that beneficiaries consider PPH as unusual blood loss followed by alteration of general health condition of the client. Participants believe that every pregnant woman has the potential to experience PPH as it happens “unexpectedly”. Among the causes of PPH commonly known as the ‘4Ts’ mnemonic: tone, tissue, trauma, and thrombin [5, 34], beneficiaries noted that PPH is associated with trauma of genital organs that might be caused by inadequate labor and delivery care or lack of birth spacing. Beneficiaries mentioned lack of balanced diet and poverty as key antenatal risk factors. Similarly Halle-Ekane et al. [35] report that poverty, lifestyle and malnutrition are some broad issues which adversely influence the outcome of a patient with PPH. It has been reported that the knowledge of contextual-level risk factors would inform public health interventions for PPH control [36].

Our results showed indication that participants had some knowledge about the consequences of excessive blood loss at childbirth. This finding concurs with Finlayson et al. [6], who conducted a qualitative systematic review appraising available evidence about the views and experiences of women and healthcare providers on interventions to prevent PPH. They found that women were generally aware of the consequences of a severe PPH, but in some situations relied on traditional healers to manage potential childbirth complications. Although our findings demonstrate that participants were generally aware of the grave consequences of PPH, some beneficiaries believed that PPH might be associated with poison, and in addition some family members (e.g. mothers-in-law) are discouraging women from accessing care at a health facility by recommending traditional medicine instead. Cultural norms around childbearing and supernatural beliefs about PPH remain a challenge in some low- and-middle income countries to address PPH prevention [37]. In addition Semasaka et al. [13] report high prevalence of poor sexual and reproductive health among Rwandan women in the early postpartum period. Education programs designed to increase awareness of the causes of PPH and the potential dangers may improve understanding in these contexts [6, 38].

Apart from the understanding about PPH causes and consequences, other factors that matter to beneficiaries in connection with prevention of PPH include avoidance of “heavy activities”, and the prevention of psychological distress associated with unsupportive partners. The emotional impact of PPH is sometimes not given much consideration in the literature but research suggests that, for some women, the repercussions can be severe and associated with long-term mental health problems including posttraumatic stress disorder [39]. Beneficiaries reported a barrier of living with low socioeconomic conditions which sometimes lead to delay in accessing health facilities. Previous research suggests that funding agencies can help underwrite initiatives aimed at reducing PPH through the use of cost-effective, resource appropriate interventions to facilitate all communities especially from low- and-middle income countries, to access quality services in a timely manner [7, 10, 37].

With regards to the prevention of PPH, our results reveal that CHWs had limited knowledge. This is in agreement with findings from a recent systematic review [40] highlighting the risk associated with inconsistent community knowledge regarding dosage and timings of misoprostol use for PPH prevention, and inconsistency of CHWs’ knowledge to differentiate between PPH caused by atony or due to other causes such as uterine rupture, vaginal lacerations and placental abnormalities. Educational sessions for all people involved in the prevention of PPH has been proposed as a way to contribute to early detection of PPH risk factors.

Our findings demonstrate that poor interaction between beneficiaries and health care providers, which can lead the women to be reluctant to express freely their health needs during childbearing period. Beneficiaries expressed feelings of frustration and anxiety when they have to enter into relationships with angry and irritable health care providers which may create a barrier to the good communication needed for the prevention of PPH. The heavy workload of health care providers was acknowledged by beneficiaries as contributing to poor communication. Our findings concur with Bohren et al. [41] confirming that overcrowded and understaffed maternity wards fostered a high-stress work environment. Improving quality of maternity care at health facilities and community settings, including women’s experiences of care, has been highlighted in recent studies [42, 43] as a key component of strategies to further reduce maternal mortality and morbidity related to PPH. It has been also noted that women must be given a platform to voice their experiences of care [41]. Particular attention can be given to patients and family seeking information about PPH and hence be a partner in their own care. Enhancing patient centred care and partnership is in accord with van der Pol et al. [44] stressing that maternity services users’ views and preferences should be taken into account in the provision of healthcare.

Our findings demonstrate that women and their relatives have trust in CHWs. The beneficiaries view the role of CHWs in charge of maternal health as being their “*parents*” in the community. This might be associated to close follow up and care by CHWs in charge of maternal health of women of reproductive age in the community. The trust in CHW’s has been also felt by midwives in a qualitative study conducted in Myanmar, as one of the supportive reasons for successful shifting basic tasks to auxiliary midwives like administration of oral misoprostol [4].

### Health worker perspectives

Our findings suggest that health care providers generally recognize PPH based on a visual estimation of blood loss among women after birth which is defined as blood loss of more than 500 ml within the first hour after birth which is in accordance with the recommended definition of the WHO [5, 7] and implemented by Rwanda Ministry of Health [45]. The Royal College of Obstetricians and Gynecologists (RCOG) in the UK recently created an amendment to this common definition of PPH. Considering that the formal measurement of postnatal blood loss suggests that this volume of blood loss is very common, occurring in up to 50% of deliveries, the RCOG approved that the postpartum blood loss of 500 ml is used as a point of ‘alert’, whereas treatment is only given once the women has lost 1000 ml of blood [34]. For CHWs in charge of maternal health included in this study, they recognize a woman with PPH when she changes the sanitary pad two or more times within the first hour following birth. Our study provides support to the guidelines developed by Rwanda Ministry of Health indicating that CHWs are required to provide close follow up of pregnant women in the community during the antenatal and postpartum periods [46]. In a situation where there are still debates on the importance of estimation or measurement of blood loss, which require particular attention for this practice, the consideration of women at risk of developing PPH may improve outcomes by early identification and timely action to mitigate risks.

Regarding PPH prevention at the community level, CHWs in this study reported adherence to the task shifting policy with administration of Misoprostol in case of a home delivery. The use of Misoprostol by CHWs in community settings has been discussed in literature [6, 7]. The shortage of well-trained health workers is global but intensified in low- and middle-income countries whereby the WHO recommends shifting basic tasks from higher- to lower-trained cadres, such as Community Health Workers or auxiliary midwives [47]. This policy is in agreement with a host of literature [6, 7] demonstrating the effectiveness of the use of Misoprostol in reducing PPH in a variety of community-based settings. However, careful attention must be paid in the use of Misoprostol in home births, especially in Rwandan settings. CHWs in charge of maternal health and some partners to the pregnant woman reported that their role is specifically to get the woman to the hospital for delivery which is similar to the findings of a study conducted in Mozambique [48]. This is in line with the guideline of Rwanda Ministry of Health stating that CHWs in charge of maternal health during their home visits, are to assist the mother with birth preparedness ,to identify danger signs and make appropriate referral [25, 46, 49]. When delivery happens at home or in transit to the health facility, CHWs are authorized to administer Misoprostol for PPH prevention, then to continue to accompany the woman to the nearest health facility for further assessment and follow up. This is contrary to some other settings of low- and-middle income countries where home deliveries are happening in the community with assistance of traditional birth attendants or auxiliary midwives [50–53].

From an organizational perspective our findings indicate that healthcare providers practice active management of the third stage of labor with injectable Oxytocin to prevent PPH, but its effectiveness is questionable as most health facilities lack refrigerator storage in the maternity units to keep oxytocin which requires transport and storage at 2 °C–8 °C regardless of the label [7]. Smith et al. [54] point out that oxytocin as an essential medicine for preventing PPH, requires proper storage with regular supply of the medicine. This suggests a need for better supply chain management of maternal health medicines and supplies, as well as greater coordination between clinical/service provision [7, 54]. Bartlett et al. [55] recommend regular training to improve active management of the third stage of labor optimizing the timing of uterotonic administration.

## Strengths and limitations to the study

A strength of our study is its approach of triangulation [56] used to obtain credible information on influencing factors to the provision of quality care for PPH prevention. We used different methods and different sources to collect data to develop a comprehensive understanding of the phenomenon under study from health facilities of different levels of the health system of Rwanda. Another strength is use of the multidisciplinary approach, including all health professionals involved in PPH prevention care (medical doctors, nurses and midwives), CHWs in charge of maternal health, and beneficiaries, women who experienced PPH and their close relatives including their partners. We organized one to one interviews with women and FGDs with other participants to identify influencing factors for early detection of women with PPH and its prevention from both beneficiary and health worker perspectives. This aligns with Kumar [57] recommendation to address the needs at individual, community and political levels to promote maternal health and to reduce the burden of maternal morbidity and mortality due to PPH. Our findings support the using of social determinants approach [19]to PPH prevention.

Some study limitations bear mentioning. The factors influencing the prevention of PPH and early detection of women at risk described by participants in the present study are limited to personal views and have been analyzed qualitatively. Therefore, a further case control study using quantitative methods is warranted to analyze predictors for PPH among childbearing women in Rwanda. The general applicability of our findings may be questionable as the selected health facilities were from a rural area and thus may not be applicable to the health facilities from urban areas. Nevertheless, our results apply to international guidelines for PPH prevention [5, 7] because the guidelines were referred to develop our interview guides.

## Conclusions

In conclusion, influencing factors for for prevention of PPH and early detection of women at risk were described, from both beneficiary and health worker perspectives. For beneficiaries, factors hampering PPH prevention were described to be primarily poor understanding and knowledge of PPH, while from the health worker perspective there was a focus on organizational factors associated with shortage of staff, poor team communication and collaboration and lack of refrigeration storage in maternity settings to keep injectable oxytocin nearby delivery rooms. Innovative solution to have a medication of the same quality as oxytocin but without the constraints related to heat sensitivity during the transportation and storage would be considered for PPH prevention. Development of strategies for early detection of women at risk of PPH, regular trainings of health care providers, developing educational material for CHWs and family members could improve the prevention of PPH. Further quantitative research, using case control design is warranted to develop a screening tool for early detection of PPH risk factors for a proactive prevention.

## Data Availability

The datasets used and analyzed during the current study are available from the corresponding author on reasonable request.

## Abbreviations

PPH: Postpartum hemorrhage
CHW: Community health worker
W: Woman
R: Relative
HCP: Health care provider
IDIs: Individual in-depth interviews
FGDs: Focus group discussions
